# Linear-Range Extension for Linear Variable Differential Transformer Using Hyperbolic Sine Function

**DOI:** 10.3390/s22103674

**Published:** 2022-05-12

**Authors:** Apinai Rerkratn, Jakkapun Tongcharoen, Wandee Petchmaneelumka, Vanchai Riewruja

**Affiliations:** School of Engineering, King Mongkut’s Institute of Technology Ladkrabang, Ladkrabang, Bangkok 10520, Thailand; apinai.re@kmitl.ac.th (A.R.); 61601186@kmitl.ac.th (J.T.)

**Keywords:** inductive transducer, linear variable differential transformer, linear range extension, hyperbolic sine function, class AB bipolar amplifier

## Abstract

In this paper, a circuit technique to extend the measuring range of a linear variable differential transformer (LVDT) is proposed. The transfer characteristic of the LVDT contains the odd function form of the cubic polynomial. Therefore, the measuring range of a commercial LVDT is linear in a narrow range compared to its physical dimensions. The wide measuring range of the LVDT requires a large structure of the LVDT, which increases the scale and the cost of the measurement system. The measuring range of the LVDT can be linearly extended to the maximum of the stroke range using the proposed technique. The realization of the proposed technique is based on the use of the hyperbolic sine (sinh) function of the electronic circuit building block, named the class AB bipolar amplifier. The class AB bipolar amplifier can be obtained by the current feedback operational amplifier (CFOA). The circuit of the proposed technique requires two CFOAs and an operational transconductance amplifier (OTA) as the active devices and all devices used in the proposed technique to synthesize the sinh function are commercially available. The proposed technique exhibits an ability to compensate for the nonlinear characteristic of the LVDT without digital components. The proposed technique is attractive in terms of its simple circuit configuration, small size, and low cost. The linear range extension of the LVDT used in this paper is significantly increased with a maximum error of about 18.3 μm of 6.2 mm at the full stroke range or the full-scale percentage error of about 0.295%. The results indicate that the proposed technique provides excellent performance to extend the measuring range of the LVDT without modifying the LVDT structure.

## 1. Introduction

An inductive transducer, known as the linear variable differential transformer (LVDT), is an important transducer used in a position measurement system. The LVDT provides excellent behavior in terms of high resolution, robustness, and durability [1,2,3]. Many applications of the LVDT are found in the fields of industrial process control [3,4,5,6], building construction [7], automobiles [8,9], military equipment [10,11,12], scientific and medical equipment [12,13], and robotics [14,15]. All applications of the LVDT are used to measure position, force, flow, displacement, and pressure. The LVDT structure is similar to a transformer which comprises one primary winding and two secondary windings with a moveable core. Two secondary windings of the LVDT are connected in series in opposite directions. Therefore, the resulting signal from the secondary windings is the difference signal which calls the LVDT signal. The excitation signal is applied to the primary winding and then the secondary signal or the LVDT signal is in the form of balance modulation or amplitude modulation with a suppressed carrier (AMSC) [1,2,15]. The amplitude of the LVDT signal is dependent on the core position. Therefore, a synchronous demodulator is required to extract the core position signal from the LVDT signal. There are several synchronous demodulators to obtain the core position signal. Traditionally, the simple synchronous demodulators are provided by an analog multiplier and low-pass filter, or analog switch and integrator have been proposed to extract the core position signal from the LVDT signals [1,3,15,16,17]. The disadvantage of the simple demodulator is that the large response time and phase shift of the low-pass filter or integrator degrade the accuracy of the demodulated signal. An alternative demodulation technique using a peak-amplitude finder was proposed to overcome the disadvantage mentioned above [18,19,20]. This technique exhibits a simple configuration, fast response, and high accuracy. However, the core position signal obtained from the demodulator is narrow due to the structure of the LVDT [2,21,22,23]. The large measuring range of the LVDT requires a huge structure for the LVDT, which causes an inconvenience for the applications of compact scale of the measurement system. If the linear measuring range of the LVDT can be extended without disturbing the LVDT structure, then the advantage will be gained.

The measuring range of the LVDT offers a narrow linear range due to its nonlinear transfer characteristic. The transfer characteristic of the LVDT can be expressed in the form of the odd function of the cubic polynomial, which corresponds to the first-order and the third-order terms of the series of an inverse hyperbolic sine function [2,22,23,24]. Therefore, the transfer characteristic of the LVDT can be approximated to the inverse hyperbolic sine function for its full-stroke range. It can be seen that the linear measuring range of the LVDT is linear only in the range around the zero-crossing of its transfer characteristic curve. The linear measuring range of the LVDT can provide a wide measuring range to meet such a requirement by increasing the structure size of the LVDT, which results in the bulky structure of the LVDT [21]. The large structure of the LVDT is limited only to the applications of the LVDT due to the large configuration of the measurement system. The small structure of the LVDT with a wide measuring range requires the most attention for the small or compact scale of the position measurement system. However, the requirement of the small structure of the LVDT is in contrast to the wide measuring range. The extension of the linear measuring range of the LVDT requires the most attention for reducing the configuration of the measurement systems and increasing its cost-effectiveness. Recently, a technique based on the fractional-order LVDT for extension of the linear measuring range was proposed in the literature [25]. However, the LVDT used in this technique requires a special design, which is inappropriate for a commercial LVDT. There are many techniques to extend the linear measuring range without disturbing the LVDT structure [22,23,26,27,28]. The approaches for the extension of the linear measuring range using an artificial neural network are introduced [26,27]. These techniques use an adaptive inverse model to compensate for the nonlinear transfer characteristic of the LVDT. The disadvantage of these techniques is that a high-speed processor is required to determine the adaptive inverse model. This causes a large circuit configuration, large response time, and increased expense. An alternative approach for the extension of the LVDT linear range using a binomial series of the LVDT inverse transfer characteristic is proposed in the literature [22,23]. The advantage of this technique is that a wide linear range and high accuracy are obtained. However, this technique requires precision analog multipliers to synthesize the inverse transfer characteristic of the LVDT, which requires high production cost. Additionally, the technique based on the hyperbolic tangent function to enhance the LVDT linear range is presented [28]. This technique requires two well-matched diodes and the operational amplifiers (opamp) to accurately generate the hyperbolic tangent function. Unfortunately, it is impractical to specify two well-matched diodes in both discrete and integrated forms. In addition, an overview of the literature review for the linear range extension of the LVDT is shown in Table 1.

The purpose of this paper is to propose a simple circuit for the linear range extension of the LVDT using a commercially available device. The linear range of the LVDT is extended using the inverse function of the LVDT transfer characteristic. The hyperbolic sine (sinh) function is used to compensate for the nonlinear transfer characteristic of the LVDT. The proposed technique utilizes the behavior of a current feedback operational amplifier (CFOA) with the class AB input stage to generate the sinh function. The linear range of the LVDT can be extended to its maximum stroke range, limited by LVDT’s physical dimension. The performance of the proposed technique was analyzed and discussed in detail. Experimental results exhibiting the performance of the proposed technique are included. The maximum full-scale error is about 0.295% for the core varied to the maximum stroke of the LVDT used in this paper. The proposed technique obtains the goal of a simple circuit configuration, high accuracy, and low cost.

## 2. Principle of LVDT

**Figure 1:**
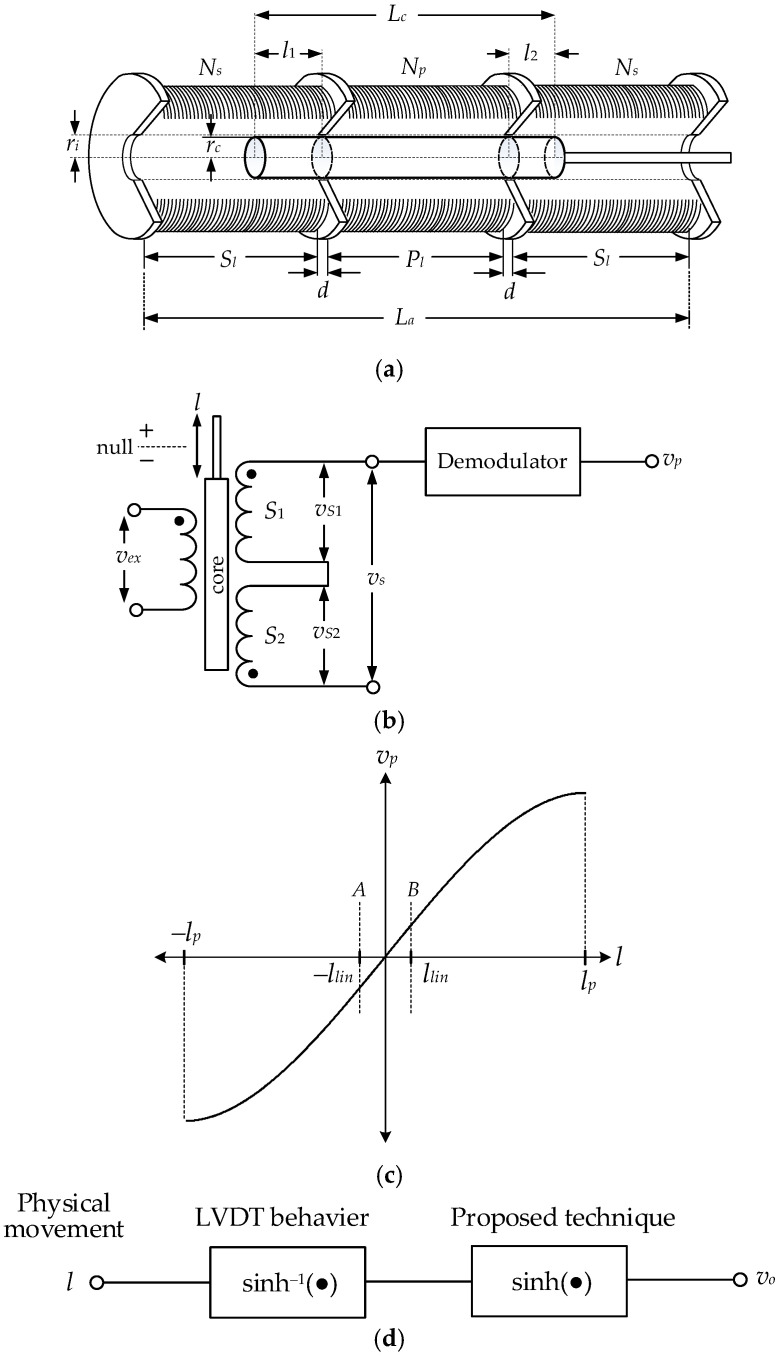
Principle of LVDT: (**a**) structure; (**b**) simplified diagram; (**c**) transfer characteristic; (**d**) block diagram for range extension.

The structure of the LVDT comprised a primary winding and two identical secondary windings curving around a hollow rod with a radius *r_l_* is shown in Figure 1a. From Figure 1a, the primary winding is placed in the middle of two secondary windings connected in opposite directions. The simplified diagram of the LVDT is shown in Figure 1b. The lengths of the primary winding and two identical secondary windings are given by *P_l_* and *S_l_*, respectively. *N_p_* and *N_s_* are the numbers of turns for primary winding and secondary winding, respectively. The moving core of the LVDT is provided from a ferromagnetic with a radius and length set as *r_c_* and *L_c_*, respectively. The gaps isolated between both sides of the primary winding and two secondary windings are assigned as *d*. When the excitation signal *v_ex_* = *V_P_*sin(*ω_ex_t*), it is applied to the primary winding. The secondary winding signals *v_S_*_1_ and *v_S_*_2_ are generated, which depend on the position of the moving core and can be stated as [2,22,23] follows:

(1) $$v_{S1}=\frac{2\pi^{2}\omega_{ex}v_{ex}N_{p}N_{s}\left(2l_{2}+P_{l}\right)}{10^{7}S_{l}L_{c}Z_{P}\ln\left(\frac{r_{l}}{r_{c}}\right)}l_{1}^{2},$$

and

(2) $$v_{S2}=\frac{2\pi^{2}\omega_{ex}v_{ex}N_{p}N_{s}\left(2l_{1}+P_{l}\right)}{10^{7}S_{l}L_{c}Z_{P}\ln\left(\frac{r_{l}}{r_{c}}\right)}l_{2}^{2}$$

where *Z_P_* is an impedance of the primary winding, *l*_1_ and *l*_2_ denote the distances for the moving core penetrated the secondary windings *S*_1_ and *S*_2_, respectively. Thus, the LVDT signal *v_s_* is equal to the difference of the secondary winding voltages, (*v_S_*_1_ – *v_S_*_2_), and can be expressed as follows:

(3) $$v_{s}=K_{1}l(1-K_{2}l^{2}),$$

for

$$K_{1}=\frac{8\omega_{ex}v_{ex}N_{p}N_{s}\left(P_{l}+2d+l_{0}\right)l_{0}}{10^{7}S_{l}L_{c}Z_{P}\ln\left(\frac{r_{l}}{r_{c}}\right)},$$

and

$$K_{2}=\frac{1}{\left(P_{l}+2d+l_{0}\right)l_{0}},$$

where *l*_0_ = (*l*_1_ + *l*_2_)/2 is the position of the moving core at the center and *l* = (*l*_1_ − *l*_2_)/2 is the distance or the position of the moving core moved from the center. Normally, the gaps *d* are very small compared to the length *P_l_* or *P_l_* >> *d*. Assigning the length of the moving core *L_c_* = (3*P_l_* + 2*d*), the LVDT signal *v_s_* can be expressed as follows:

(4) $$v_{s}=\frac{8\pi^{2}\omega_{ex}v_{ex}N_{p}N_{s}}{10^{7}Z_{P}\ln\left(\frac{r_{o}}{r_{i}}\right)}\frac{2P_{l}}{3S_{l}}\left(l-\frac{l^{3}}{2P_{l}{}^{2}}\right)=k_{se}l\left(1-k_{nl}l^{2}\right),$$

where *k_se_* and *k_nl_* are the sensitivity and the nonlinear coefficient of the LVDT, respectively. The peak amplitude of the LVDT signal *v_s_* of Equation (4) is dependent on the position *l* of the moving core in the form of AMSC. The peak amplitude of the LVDT signal *v_s_* is extracted to obtain the position signal *v_p_* by the synchronous demodulator as shown on the right of Figure 1b. The transfer characteristic of the LVDT is shown in Figure 1c for the LVDT core varied in full range. From Equation (4), the term of *k_nl_* limits the linear measuring range of the LVDT. The linear measuring range *l_lin_* can be expressed as follows:

(5) $$l_{lin}=\pm \sqrt{\frac{\varepsilon_{li}}{k_{nl}}},$$

where *ε_li_* denotes the linearity error of the LVDT. Practically, the percentage error *ε_li_* is about 0.5% for the LVDT used in this paper. The linear measuring range *l_lin_* of about 2.54 mm is determined from Equation (5) for *k_nl_* = 7.75 × 10^−4^. From Figure 1c, the stroke range ±*l_p_* of the LVDT for the peak-to-peak amplitude in the transfer characteristic can be given as follows:

(6) $$l_{p}=\pm \sqrt{\frac{1}{3k_{nl}}}.$$

In addition, the stroke range ±*l_d_* of the LVDT due to its physical dimensions can be determined from the lengths *L_a_* and *L_c_* of the structure in Figure 1a as follows:

(7) $$\pm l_{d}=\pm \frac{\left(L_{a}-L_{c}\right)}{2}.$$

It should be noted that the maximum stroke range *l_max_* of the LVDT is limited by the minimum number between the stroke ranges *l_p_* and *l_d_* and can be written as follows:

(8) $$l_{max}=\pm \min\left(l_{d},l_{p}\right).$$

From Equation (7), the lengths *L_a_* and *L_c_* for the physical dimensions of the LVDT used in this paper are equal to 44.1 mm and 31.8 mm, respectively. Therefore, the maximum stroke range ±*l_max_* for the LVDT used in this paper is equal to ±*l_d_* = ±6.2 mm. From Equation (5), if the term of *k_nl_* is decreased, then the linear measuring range of the LVDT is achieved for a wide range. Unfortunately, the small value of *k_nl_* can be obtained by increasing the length and the number of turns for the primary winding, which causes the large dimensions of the LVDT structure. The LVDT signal *v_s_* in Equations (3) and (4) is the odd function form of the cubic polynomial, which corresponds to the series of the inverse hyperbolic sine (sinh^−1^) function for the first- and third-order terms. The series of the sinh^−1^ function is given by [24] as follows:

(9) $$\sinh^{-1}x=x-a_{1}x^{3}+a_{2}x^{5}-a_{3}x^{7}+\ldots \ldots \;\mathrm{for}\;-1<x<1,$$

where

$$a_{n}=\sum_{n=1}^{\infty }\left[\frac{\left(2n\right)!}{2^{2n}\left(2n+1\right){\left(n!\right)}^{2}}\right]$$

From Equation (9), the magnitudes of the terms *a_n_x*^(2*n*+1)^ for *n* ≥ 2 are very small and can be ignored. Thus, the position signal *v_p_* demodulated from the LVDT signal *v_s_* in Equation (4) can be approximated as follows:

(10) $$v_{p}=\frac{k_{se}}{\lambda }\sinh^{-1}\left(\lambda l\right)$$

where *λ* is the scaling factor to maintain the condition of −1 < *λl* < 1 for the core of the LVDT varied in the maximum stroke range. The scaling factor *λ* can be determined from the maximum error of the LVDT in its linear measuring range and the magnitude of the sinh^−1^ function in Equation (10) at the maximum error *ε_max_* of the LVDT signal in Equation (4). Therefore, the scaling factor *λ* can be given approximately as follows:

(11) $$\lambda =\frac{\sqrt{3!\left(\varepsilon_{max}\right)}}{l_{lin}}$$

From Equation (4), the transfer characteristic of the LVDT is nonlinear with respect to the moving core *l*. The linear range of the LVDT transfer characteristic is achieved for the core position closed to *l*_0_, which corresponds to the condition of *k_n_l*^2^ << 1. If the core position *l* is varied in the linear range of ±*l_lin_* in Equation (5). Then, the signal *v_p_* of Equation (10) can be given as follows:

(12) $$v_{p}=k_{se}l$$

It should be noted that Equation (12) is satisfied only for the core position *l* varied in the range of the dashed line A and B in Figure 1c. Unfortunately, the linear range of the LVDT is very narrow compared to its physical dimensions. The linear measurement of the LVDT for a wide measuring range causes a large structure, which limits the application of the LVDT for the small- or compact- measurement systems. To extend the linear measuring range, the sinh^−1^ function of the LVDT behavior is linearized using the sinh function circuit as shown in the block diagram of Figure 1d.

## 3. Synthesis of Sinh Function

**Figure 2:**
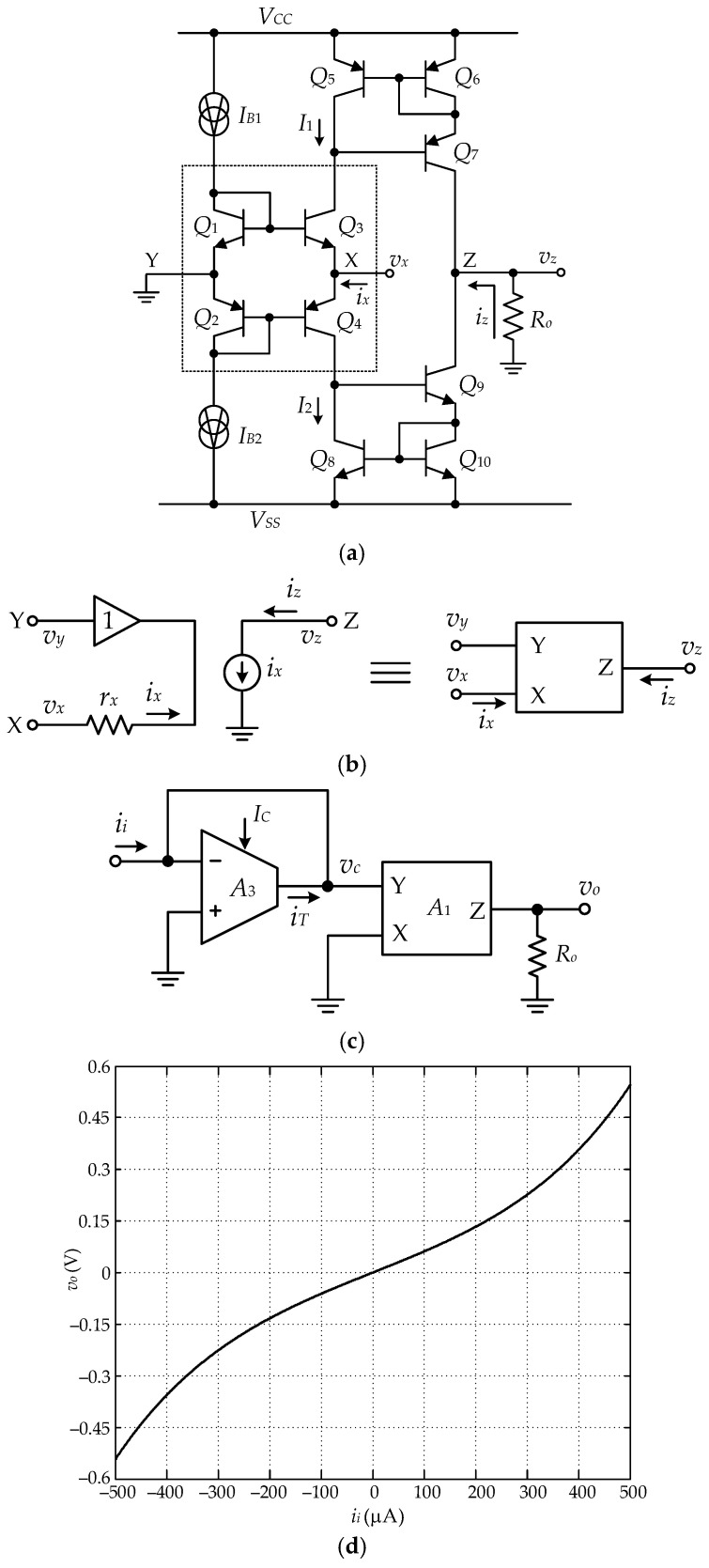
Synthesis of sinh function: (**a**) CFOA configuration; (**b**) equivalent diagram of CFOA; (**c**) sinh function circuit; (**d**) transfer characteristic of Figure 2c.

The synthesis of the sinh function utilizes the inherent behavior of the bipolar transistor-based class AB configuration. The class AB configuration can be provided from a commercially available CFOA, as shown in Figure 2a [29]. The basic principle of the bipolar class AB configuration is shown in the dash-line frame of Figure 2a. The currents *I_B_*_1_ = *I_B_*_2_ = *I_B_* are the bias current for the transistors *Q*_1_ to *Q*_4_. From Figure 2a, the currents *I*_1_ and *I*_2_ can be expressed as in [30], as follows:

(13) $$I_{1}=-\frac{i_{x}}{2}+I_{B}\sqrt{{\left(\frac{i_{x}}{2I_{B}}\right)}^{2}+1}$$

(14) $$I_{2}=\frac{i_{x}}{2}+I_{B}\sqrt{{\left(\frac{i_{x}}{2I_{B}}\right)}^{2}+1}$$

From Equations (13) and (14), the relationship between the voltage signal *v_x_* and the current *i_x_* can be given as follows:

(15) $$v_{x}=V_{T}\ln\left[\frac{i_{x}}{2I_{B}}+\sqrt{{\left(\frac{i_{x}}{2I_{B}}\right)}^{2}+1}\right]$$

where *V_T_* = *kT*/*q* is a thermal voltage, *k* = 1.38 × 10^−23^ J/K is a Boltzmann’s constant, *q* = 1.602 × 10^−19^ C is an electron charge, and *T* = (273+ °C) in Kelvin is an absolute temperature [31]. From the expression of the sinh^−1^ function, Equation (15) can be written in the form of the sinh^−1^ function as [24], as follows:

(16) $$v_{x}=V_{T}\sinh^{-1}\left(\frac{i_{x}}{2I_{B}}\right)$$

It should be noted that the voltage signal *v_x_* is dependent on temperature due to the thermal voltage *V_T_*. This temperature effect can be compensated by the simple circuit technique discussed in the next explanation. From Equation (15), the relationship of the voltage *v_x_* and the current signal *i_x_* can be rewritten as follows:

(17) $$i_{x}=2I_{B}\sinh\left(\frac{v_{x}}{V_{T}}\right)$$

From Figure 2a, the current *i_x_* can be obtained by the subtraction of the currents *I*_1_ and *I*_2_ of Equations (13) and (14), respectively. The current mirrors *CM*_1_ and *CM*_2_ formed by the transistors *Q*_5_–*Q*_7_ and *Q*_8_–*Q*_10_, respectively, transfer the currents *I*_1_ and *I*_2_ to the output port z and the current *i_z_* can be stated as follows:

(18) $$i_{z}=I_{2}-I_{1}=i_{x}$$

The output current *i_z_* can be simply converted to the voltage signal *v_z_* by the resistance *R_o_*. From Equation (16), the sinh^−1^ function can be approximated to a linear function for the condition of *i_x_* << 2*I_B_*. Thus, Equation (16) can be given as follows:

(19) $$v_{x}=\frac{V_{T}}{2I_{B}}i_{x}=r_{x}i_{x}$$

where *r_x_* denotes a small-signal resistance at port x of the CFOA. The schematic diagram of the CFOA in Figure 2a can be represented by the equivalent diagram as shown in Figure 2b. The circuit diagram for synthesizing the sinh function is shown in Figure 2c. An operational transconductance amplifier (OTA), *A*_3_, acting as an active resistor, is provided to compensate for the thermal voltage *V_T_*. The output current *i_T_* of the OTA *A*_3_ can be given as follows:

(20) $$i_{T}=-\frac{I_{C}}{2V_{T}}v_{c}$$

where *I_C_* denotes the bias current of the OTA *A*_3_. From the circuit in Figure 2c, the input current *i_i_* is equal to the current *i_T_* with the opposite direction, or *i_i_* = −*i_T_*. Therefore, the voltage signal *v_c_* across the input of the OTA *A*_3_ is equal to (2*V_T_/I_C_*)*i_i_*, which is provided for the input signal of the sinh function formed by the CFOA *A*_1_. From Equations (17) and (20), the relationship between the output voltage signal *v_o_* and the input current signal *i_i_* can be stated as follows:

(21) $$v_{o}=2I_{B}R_{o}\sinh\left(\frac{2}{I_{C}}i_{i}\right)=k_{o}\sinh\left(\frac{2}{I_{C}}i_{i}\right)$$

It should be noted that the temperature effect due to the thermal voltage *V_T_* is compensated for. The input signal of the circuit in Figure 2c is in the form of a current signal. Thus, the voltage to current converter is required to convert the position signal *v_p_* into the current form. Figure 2d shows the simulation result of the circuit in Figure 2c, where *I_B_* = 211.88 μA, *I_C_* = 500 μA, and *R_o_* = 3.46 kΩ.

## 4. Proposed Circuit for LVDT Linear Range Extension

**Figure 3:**
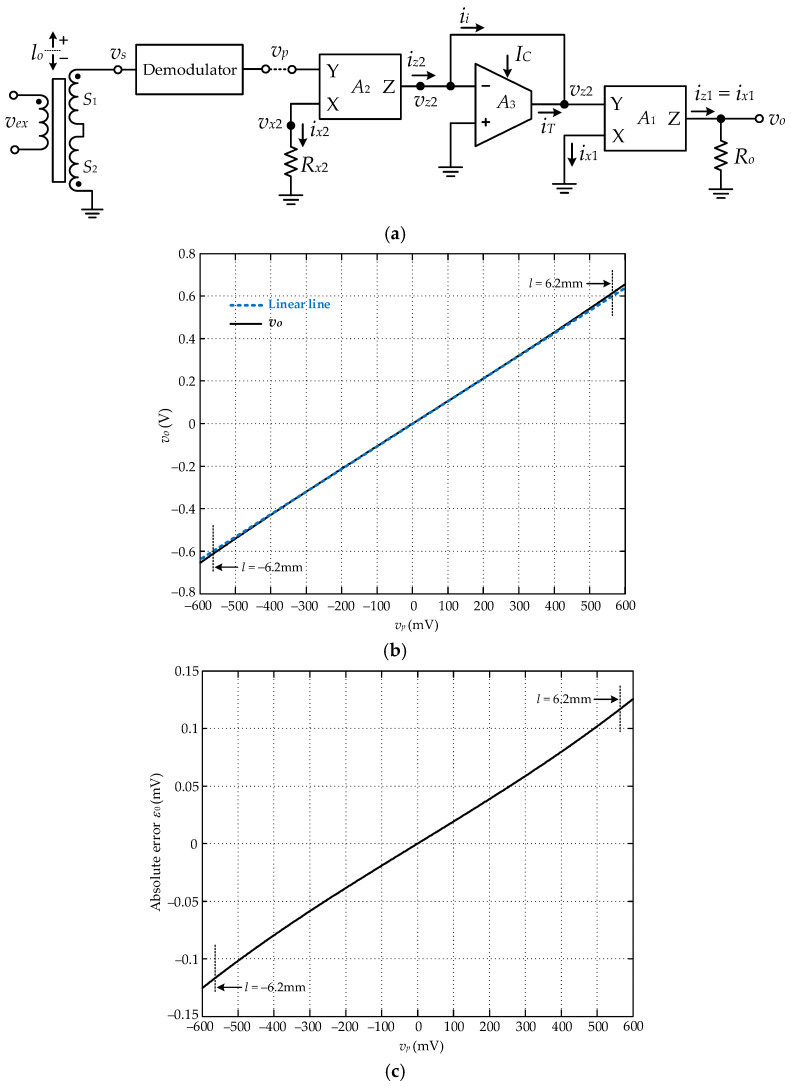
Proposed circuit: (**a**) circuit diagram; (**b**) simulation result; (**c**) absolute error *ε_o_*.

The proposed circuit for the extension of the LVDT linear measuring range is shown in Figure 3a. The operation of the circuit can be explained as follows. The demodulated signal *v_p_* is transferred to the voltage signal *v_x_*_2_ at port x of the CFOA *A*_2_, which can be stated as follows:

(22) $$v_{x2}=\frac{R_{x2}}{R_{x2}+r_{x2}}v_{p}=\frac{gm_{c2}R_{x2}}{gm_{c2}R_{x2}+1}v_{p}$$

where *gm_c_*_2_ = 2*I_B_*/*V_T_* denotes the transconductance at port x of the CFOA *A*_2_ and *r_x_*_2_ = 1/*gm_c_*_2_ = *V_T_*/2*I_B_*. The bias current *I_B_* is about 211.88 μA for the CFOAs used in this paper. The thermal voltage *V_T_* at 25 °C is about 25.69 mV and the resistance *r_x_*_2_ is about 60.62 Ω is calculated from Equation (19). If the resistance *gm_c_*_2_*R_x_*_2_ >> 1 is assigned, then the voltage signal *v_x_*_2_ can be approximately equal to the position signal *v_p_*. From Figure 3a, the current *i_x_*_2_ flowing through the resistance *R_x_*_2_ is equal to *v_p_*/*R_x_*_2_ and transferred to the current *i_z_*_2_ at port z of the CFOA *A*_2_, *i_z_*_2_ = *i_x_*_2_. The current *i_z_*_2_ is provided for the input current of the OTA *A*_3_ to generate the voltage signal *v_z_*_2_. Thus, the voltage signal *v_z_*_2_ can be expressed as follows:

(23) $$v_{z2}=\frac{v_{p}}{gm_{c3}R_{x2}}=\frac{2V_{T}}{I_{C}R_{x2}}v_{p}$$

The voltage signal *v_z_*_2_ is applied to port y of the CFOA *A*_1_. From Equations (10), (21), and (23), the voltage signal *v_o_* can be given as follows:

(24) $$v_{o}=\frac{k_{o}}{\lambda }\sinh\left[\frac{2k_{se}\sinh^{-1}\left(\lambda l\right)}{\lambda I_{C}R_{x2}}\right]$$

To linearize the LVDT behavior in Equation (10), the position of the LVDT core at *l* = *l_lin_* is used as the reference position. The magnitude of the sinh function in Equation (24) is assigned to equal the demodulated signal *v_p_* in Equation (12) with the core position *l* = *l_lin,_* as follows:

(25) $$\frac{k_{o}}{\lambda }\sinh\left[\frac{2k_{se}\sinh^{-1}\left(\lambda l_{lin}\right)}{\lambda I_{C}R_{x2}}\right]=k_{se}l_{lin}$$

From Equation (25), the terms of 2*k_se_*/(λ*I_C_R_x_*_2_) = 1 should be fulfilled, and *k_o_* = *k_se_* is set for the sensitivity of the proposed circuit as the same as the LVDT. Therefore, the voltage signal *v_o_* in Equation (24) is equal to *k_se_l* for the core position *l* varied in the maximum range ±*l_max_.* The relationship of the resistance *R_x_*_2_ and the bias current *I_C_* of the OTA *A*_3_ can be stated as follows:

(26) $$R_{x2}=\frac{2k_{se}}{\lambda I_{C}}=\frac{2k_{se}l_{lin}}{I_{C}\sqrt{3!\left(\varepsilon_{max}\right)}}$$

The coefficient *k_o_* in Equation (24) can be set to meet the required sensitivity by tuning the resistance *R_o_*. The resistance *R_o_* can be determined from the required sensitivity *k_req_* as follows:

(27) $$R_{o}=\frac{k_{req}}{2\lambda I_{B}}$$

If the sensitivity *k_req_* of the proposed circuit is assigned as 0.1 V/mm/V, then the resistance *R_o_* = 3.46 kΩ is calculated from Equation (27) for *I_B_* = 211.88 μA.

The LVDT used for this paper provides sensitivity *k_se_* of 94.5 mV/mm/V a the nonlinear coefficient *k_nl_* of 7.75 × 10^−4^. The input signal *v_p_* of Figure 3a is linearly varied in the range ±0.5684 V, which corresponds to the LVDT core linearly varied in the range ±6.2 mm. This input signal is used for the simulation to investigate the transfer characteristic of the proposed circuit via PSPICE analog simulation program. The simulation result for the transfer characteristic of the proposed circuit in Figure 3a is shown in Figure 3b, where λ = 0.068, *R_x_*_2_ = 5.542 kΩ, *R_o_* = 3.46 kΩ, *I_B_* = 211.88μA, and *I_C_* = 500 μA. The output signal error *ε_o_* is obtained by subtracting the expected value, the linear line in Figure 3b, from the output signal *v_o_*. Figure 3c shows the absolute error *ε_o_* from the expected value of the output signal *v_o_*. The maximum error *ε_o_* is about 0.12 mV, corresponding to 0.12μm at the input signal *v_p_* = ±0.5684 V, corresponding to the core position *l* = ±*l_max_* = ±6.2 mm. It is confirmed that the proposed circuit can accurately extend the linear range of the LVDT, in agreement with the theoretical expectation.

## 5. Performance Analysis

The performance of the proposed circuit can be disturbed by the nonideal characteristic of the devices used in the scheme. There are three major factors that cause the deviation of the ideal performance. Firstly, the finite resistance *r_x_*_2_ of the CFOA *A*_2_ in Figure 3a causes the transfer error *ε_x_* in the current signal *i_z_*_2_. From Equation (22), the current signal *i_z_*_2_ = *i_x_*_2_ = *v_x_*_2_/*R_x_*_2_ is obtained for the condition of *R_x_*_2_ >> *r_x_*_2_. However, the relationship between the current signal *i_z_*_2_ and the voltage signal *v_p_* can be written as follows:

(28) $$i_{z2}=\frac{v_{p}}{\left(R_{x2}+r_{x2}\right)}=\frac{v_{p}}{R_{x2}}\left(1-\varepsilon_{x}\right)$$

and

(29) $$\varepsilon_{x}=\frac{r_{x2}}{\left(R_{x2}+r_{x2}\right)}$$

If the resistances *R_x_*_2_ and *r_x_*_2_ are set as 5.542 kΩ and 60.62 Ω, respectively, then a transfer error *ε_x_* of about 1.08% is predicted. Secondly, the variation of the ambient temperature causes the derivation in the current *i_z_*_2_ due to the thermal voltage *V_T_*. The percentage error *ε_z_* of the current *i_z_*_2_ can be stated as follows:

(30) $$\varepsilon_{z}=\frac{\Delta i_{z2}}{i_{z2}}=\frac{-2V_{T}}{\left(4V_{T}+I_{B}R_{x2}\right)}\frac{\Delta T}{T}\times 100\%$$

If the ambient temperature deviates 10 °C from room temperature at 25 °C, then the percentage error *ε*_z_ can be predicted as 0.019% for Δ*T* = 10 °C, *T* = 308 K. It can be seen that the variation of the ambient temperature is insignificant regarding disturbing the proposed circuit performance. Thirdly, the approximation of the LVDT transfer characteristic to the sinh^−1^ function exhibits the residual error *ε_r_*, which can be expressed in the expansion series as in [24], as follows:

(31) $$\varepsilon_{r}=\left\{\left(\frac{\lambda^{2}}{3!}-k_{n}\right)l_{lin}^{2}+\sum_{n=2}^{\infty }\left[\frac{\left(2n\right)!}{2^{n}\left(2n+1\right){\left(n!\right)}^{2}}\right]{\left(\lambda l_{lin}\right)}^{2n}\right\}\times 100\%$$

From Equation (31), the second term in the curly bracket is very small due to the value of (*λl_lin_*)^2*n*^ << 1 and can be ignored. Then, Equation (30) can be approximately given as follows:

(32) $$\varepsilon_{r}=\left(\frac{\lambda^{2}}{3!}-k_{n}\right)l_{lin}^{2}\times 100\%$$

From Equation (32), the residual error *ε_r_* is about 0.027% for the LVDT used in this paper, which is very small and can be ignored.

It should be noted that the major parameter affecting the accuracy of the proposed circuit is the transfer error *ε_x_* caused by the intrinsic resistance *r_x_*_2_ of the CFOA *A*_2_. This error can be minimized by replacing the resistance *R_x_*_2_ with the recalculated resistance *R_x_*_2*new*_ = (*R_x_*_2_ − *r_x_*_2_) or *R_x_*_2*new*_ = 5.481 kΩ, where *R_x_*_2_ = 5.542 kΩ and *r_x_*_2_ = 60.62 Ω. The variable resistor is provided for the recalculated resistance *R_x_*_2*new*_ and fine-tuned to meet the resistance of 5.481 kΩ.

## 6. Experimental and Simulation Results

**Figure 4:**
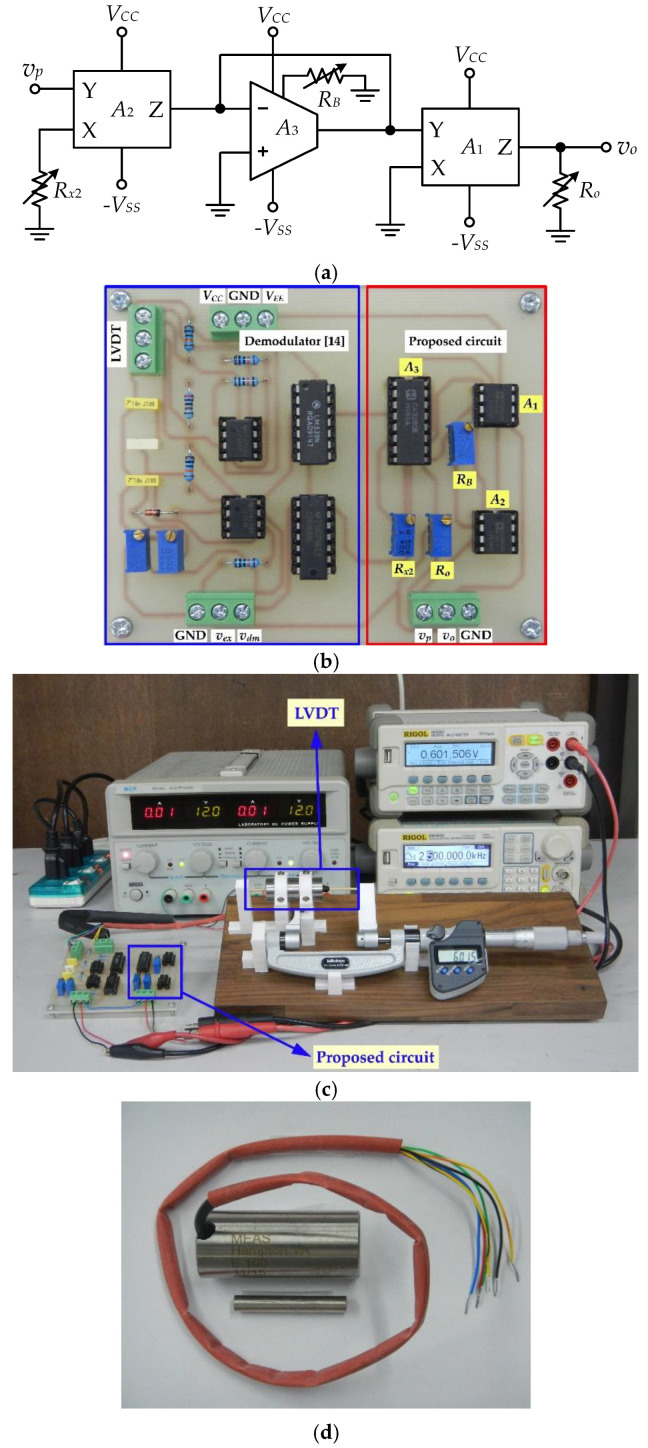
(**a**) Circuit for implementation; (**b**) prototype board; (**c**) experimental setup; (**d**) LVDT used in this paper.

The proposed circuit in Figure 3a was rearranged as shown in Figure 4a, where the resistors *R_x_*_2_ and *R_o_* are replaced with the variable resistors for convenient adjustment of the circuit parameters. The resistor *R_B_* is provided for the bias current *I_C_* of the OTA. The circuit in Figure 4a was constructed using commercial devices to investigate the performance of the proposed circuit. The active devices were AD844 for the CFOAs *A*_1_ and *A*_2_, and CA3280 for the OTA *A*_3_. The prototype board of the circuit in Figure 4a is shown in Figure 4b. The small-signal resistance *r_x_* of the CFOA was measured and calculated from Equation (19) using the equivalent circuit of AD844 as 60.62 Ω [29]. Therefore, the CFOA bias current *I_B_* was calculated from Equation (19) as 211.88 μA for the thermal voltage *V_T_* = 25.68 mV at 25 °C. The power supply voltage *V_CC_* = −*V_SS_* and the bias current *I_C_* of the OTA *A*_3_ were set as 12 V and 500 μA, respectively. The bias current *I_C_* can be achieved by the resistance *R_B_*, which is calculated from the equivalent circuit of the OTA CA3280 in [32] as follows:

(33) $$R_{B}=\frac{\left|V_{SS}\right|-1.2V}{I_{C}}$$

The resistance *R_B_* = 21.6 kΩ for the bias current *I_C_* of 500 μA is calculated from Equation (33). The power consumption of the prototype board can be calculated from the supply current and voltage of the active devices. The supply current of the CFOA and the OTA can be calculated from their equivalent circuits as 0.85 mA and 1.25 mA, respectively [29,32]. The power consumption of the CFOA and OTA of about 20.3 mW and 30 mW, respectively, for the power supply voltage *V_CC_* = −*V_SS_* = 12 V. Therefore, the power consumption of the prototype circuit is about 70.6 mW. The LVDT used in this experiment was the commercially available LVDT with the linear range *l_lin_* of ±2.54 mm, the full-scale error *ε_li_* of 0.5%, and the sensitivity *k_se_* = 94.5 mV/mm/V. The coefficient *k_nl_* = 7.75 × 10^−4^ was calculated from Equation (5). The scaling factor *λ* = 0.068 was calculated from Equation (11). The load resistance *R_L_* of the LVDT was assigned as 100 kΩ. From Figure 1a, the physical dimensions of the LVDT being used were measured as *L_a_* = 44.1 mm and *L_c_* = 31.7 mm. Therefore, the maximum stroke range *l_max_* of this LVDT is determined from Equation (7) as ±6.2 mm. The excitation signal *v_ex_* was a 2.5 kHz sinusoidal signal with 2 V peak-to-peak amplitude. The LVDT signal *v_s_* was demodulated as the signal *v_p_* using the peak-amplitude finder proposed in the literature [14]. The resistance *R_x_*_2_ is calculated from Equation (25) as 5.542 kΩ. The resistance *R_x_*_2_ is recalculated to reduce the effect of the resistance *r_x_*_2_, therefore, the resistance *R_x_*_2*new*_ = (*R_x_*_2_ − *r_x_*_2_) = 5.481 kΩ is achieved to replace the resistance *R_x_*_2_ in the proposed circuit. The sensitivity *k_req_* of the proposed circuit for this experiment is assigned as 0.1 V/mm/V. Therefore, the resistance *R_o_* is calculated as 3.46 kΩ using Equation (27). The variable resistors were provided for the resistances *R_x_*_2_ = *R_x_*_2*new*_, *R_o_*, and *R_B_* to obtain 5.481 kΩ, 3.46 kΩ, and 21.6 kΩ respectively. The experimental setup and the LVDT used in this experiment are shown in Figure 4c,d, respectively.

The full-scale error *ε_fs_* for this experiment is defined from the maximum of the absolute error and the full stroke range as follows:

(34) $$\varepsilon_{fs}=\frac{\max\left(\left|expected\;value-measured\;value\right|\right)}{maximum\;stroke\;range}\times 100\%$$

**Figure 5:**
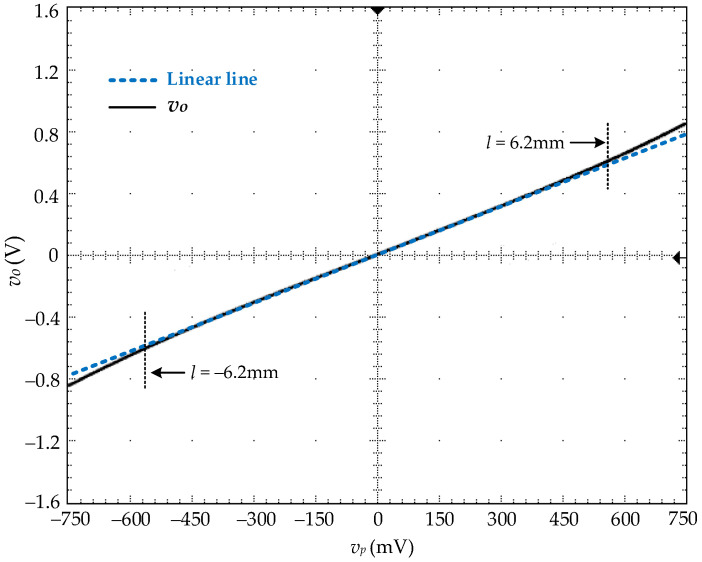
Measure transfer characteristic of prototype board.

The input signal *v_p_* of the proposed circuit in Figure 4a is linearly varied from −568.45 mV to 568.45 mV corresponding to the maximum stroke range of −6.2 mm to 6.2 mm with *k_se_* = 94.5 mV/mm/V for the LVDT being used. This input signal *v_p_* is applied to the prototype board to investigate the sinh function synthesized by the proposed circuit. Figure 5 shows the measured result of the transfer characteristic of the proposed circuit. It is evident that the proposed circuit can accurately synthesize the sinh function as expected.

**Figure 6:**
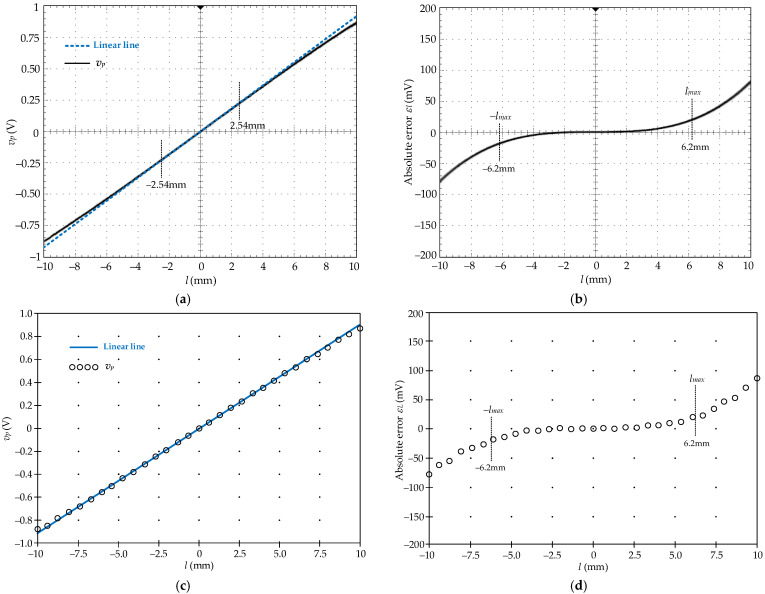
(**a**) LVDT synthesized signal; (**b**) absolute error *ε_l_*; (**c**) measured results from LVDT; (**d**) absolute error *ε_L_*.

There are two types of the input signal to demonstrate the performance of the proposed circuit, the LVDT synthesized signal and the LVDT demodulated signal. For the LVDT synthesized signal, the LVDT transfer characteristic in Equation (4) is synthesized using the LabVIEW computer program for measurement and control interfaced with analog input/output (NAIO) board from National Instruments (NI-USB-6361). The parameters *k_se_* and *k_nl_* are set to the same as the practical LVDT used in this paper. Figure 6a shows the output signal synthesized by the LabVIEW and the NAIO board. Figure 6b shows the absolute error *ε_l_* of the synthesized signal compared to the practical LVDT signal for the moving core varied from −*l_max_* to *l_max_* or −6.2 mm to 6.2 mm. From Figure 6b, the error at the core positions of ±2.54 mm and ±6.2 mm of the synthesized signal are about 12.6 μm and 184.8 μm or 1.26 mV and 18.48 mV, which correspond to 0.5% and 2.97%, respectively. For the practical LVDT signal, the LVDT was excited by the excitation signal *v_ex_.* Figure 6c,d shows the measured characteristic of the LVDT being used and the absolute error *ε_L_* from the expected value of *k_se_* × *l*, respectively. The errors of the LVDT signal at the linear range of *l* = ±2.54 mm and the maximum range of *l* = ±6.2 mm are about 0.5% and 2.98%, respectively. It can be seen that the synthesized signal in Figure 6a is closed to the LVDT transfer characteristic measured in Figure 6c. The synthesized signal in Figure 6a is provided to represent the LVDT demodulated signal *v_p_* of the proposed circuit board. The core of the LVDT can be moved over the linear range of *l* = ±*l_lin_* to the maximum position of *l* =±*l_max_*. However, the error of the LVDT demodulated signal *v_p_* is, practically, increased from 0.5% to 2.98%. This error is too high for precision measurement systems. The wide stroke range of the LVDT requires a large size for the LVDT structure, which is unsuitable for the small or compact configuration of the measurement systems. The linear range extension of the LVDT used in this paper is expected to the maximum stroke range *l_max_* as ±6.2 mm, which corresponds to the output voltage *v_o_* of ±0.62 V for the sensitivity *k_req_* = 0.1 V/mm/V. The synthesized signal from the NAIO board is applied as the input signal *v_p_* for the prototype board to demonstrate the circuit performance. The LVDT synthesized signal varied from −568.45 mV to 568.45 mV and is represented as the LVDT core continuously moving from −6.2 mm to 6.2 mm.

**Figure 7:**
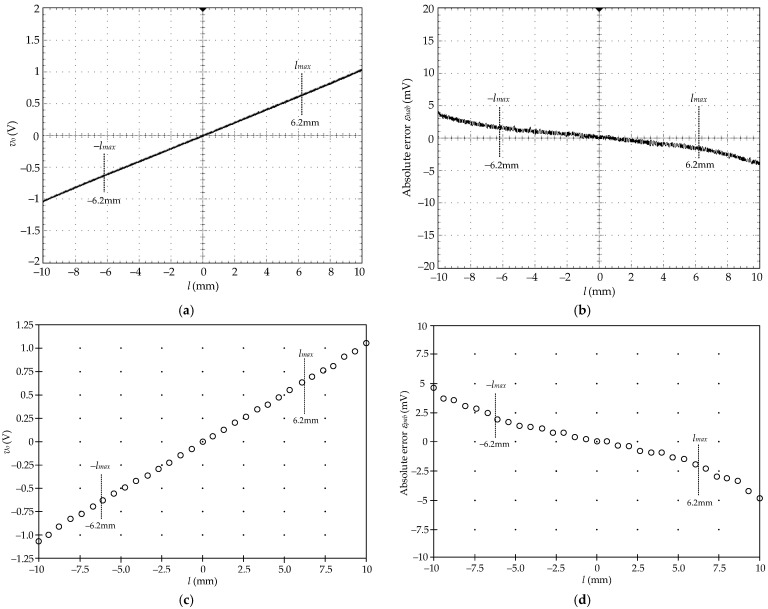
(**a**) LVDT synthesized signal; (**b**) absolute error *ε_nab_*; (**c**) measured result from LVDT; (**d**) absolute error *ε_pab_*.

Figure 7a shows the measured results of the output signal *v_o_* of the prototype board for the demodulated signal *v_p_* varied from −568.45 mV to 568.45 mV and corresponding to the LVDT core varied from −6.2 mm to 6.2 mm. The absolute error *ε_nab_* for the LVDT core varied in the range ±6.2 mm is exhibited in Figure 7b. In Figure 7b, the maximum error of about 1.6 mV at 568.45 mV or *l_max_* = 6.2 mm corresponds to the position error of 16 μm. Therefore, the full-scale error *ε_fs_* is obtained as 0.258% at the maximum stroke range. The LVDT is excited by the excitation signal *v_ex_* and the LVDT demodulated signal *v_dm_* from the peak-amplitude finder on the left of the prototype board is applied to the input signal *v_p_* on the right of the prototype board in Figure 4b. The input signal *v_p_* from the LVDT demodulated signal *v_dm_* corresponds to the LVDT core varied from −6.2 mm to 6.2 mm with 50 μm increments. Figure 7c,d shows the measured results for the LVDT core varied in the maximum range ±*l_max_* and the absolute error *ε_pab_*, respectively. It can be seen that the maximum absolute error at the core position of 6.2 mm is about 1.83 mV or 18.3 μm corresponding to the full-scale error *ε_fs_* of 0.295%. This error can be further minimized by fine-tuning the resistance *R_x_*_2_ to compensate for the intrinsic resistance of the CFOA *A*_2_ in the implementation procedure. Finally, the proposed technique can extend the linear measuring range of the LVDT from its narrow specific range to the maximum range of its physical dimensions.

The previous works proposed in the literature [22,23,26,27,28] are referenced for comparison with the implemented proposed technique in terms of percentage error, type of signal processing, complexity of implementation, production cost, and power consumption. It should be noted that the percentage errors of the previous works [26,27] are achieved only from simulation results and their power consumption is dependent on the computer system and interface board being used. Table 2 shows the comparisons between the proposed technique and those of the previous works. From Table 2, the percentage error of the proposed technique exhibits higher than that of the previous works [22,23]. This is a minor consideration compared to the remaining condition. This error can be further minimized, as mentioned in this section. The proposed circuit demonstrates its advantages in convenient implementation, low cost, low power consumption, and small error. It is evident that the proposed technique provides good performance at an attractive cost.

## 7. Conclusions

A linear range extension for a commercial LVDT using a sinh function was introduced. The sinh function was synthesized using an inherent behavior of a bipolar class AB configuration. The input stage comprises the class AB configuration of a CFOA and was utilized for the synthesis of the sinh function. All active devices used in the proposed circuit are commercially available. The advantages of the proposed circuit are its low cost and simple configuration. The performance of the proposed circuit was analyzed and discussed in detail. The linear measuring range of the LVDT used in this paper can be extended from ±2.54 mm to the maximum stroke range of ±6.2 mm. The maximum full-scale error occurred at the full stroke range of about 0.295% is achieved. It should be noted that the proposed scheme offers excellent performance in terms of high accuracy. The experimental results demonstrated that the performance of the proposed circuit agrees with the theoretical expectations.

## Figures and Tables

**Table 1:** Overview of literature review for linear range extension.

	Range Extension Technique	Requiring Equipment	System Dimension
[22]	Inverse transfer characteristic	- 2 precision analog multipliers	Small
		- 2 opamps	
[23]	Binomial series approximation	- 2 precision analog multipliers	Small
		- 1 opamp	
[26]	Artificial neural network	- High-speed computer based system	Bulky
		- Analog interface board	
[27]	Artificial neural network	- High-speed computer based system	Bulky
[28]	Hyperbolic arctangent function	- 4 opamps	Small
		- 2 well-matched diodes	

**Table 2:** Comparison between proposed technique and previous works.

	[22]	[23]	[26]	[27]	[28]	Proposed Technique
Error	0.068% (experiment)	0.23% (experiment)	0.511% (simulation)	0.294% (simulation)	2.5% (experiment)	0.295% (experiment)
Signal processing	Analog	Analog	Digital	Digital	Analog	Analog
Cost	Medium to high	Medium to high	high	high	low	low
Complexity	Medium to high	Medium to high	high	high	Medium to high	simple
Power consumption	691.2 mW	537.6 mW	N/A *	N/A *	172.8 mW	70.6 mW

* dependent on power consumption of computer system and interface board used in [26,27].

## Data Availability

Not applicable.

## Nomenclature

*ε_r_*	Residual error	*v_p_*	Position signal or demodulated signal
*ε_x_*	Transfer error	*ω_ex_*	Excitation frequency (rad/sec)
*ε_z_*	Error due to ambient temperature	*v*_*S*1_, *v*_*S*2_	LVDT secondary signal
*ε_fs_*	Full-scale error	*v_s_*	LVDT signal
*ε_li_*	Linearity error of LVDT	*Z_P_*	Impedance of primary winding
*ε_l_*	Absolute error of synthesized LVDT signal	*λ*	Scaling factor
*ε_L_*	Absolute error of practical LVDT signal	*l_o_*	Center position of moving core
*ε_o_*	Absolute error of output signal v_o_ from simulation result	*l*_1_, *l*_2_	Distance of moving core penetrated secondary windings
*ε_nab_*	Absolute error of output signal v_o_ for synthesized LVDT signal	*l_d_*	Stroke range of LVDT due to physical dimension
*ε_pab_*	Absolute error of output signal v_o_ for practical LVDT signal	*l_p_*	Stroke range for peak-to-peak amplitude of LVDT transfer characteristic
*ε_max_*	Maximum error of LVDT	*l*	Moving core position deviated from l_o_
*P_l_*	Length of the primary winding	*l_max_*	Maximum stroke range of LVDT
*S_l_*	Length of the secondary winding	*k_se_*	Sensitivity coefficient of LVDT
*N_p_*	Number of turns for primary winding	*k_nl_*	Nonlinear coefficient of LVDT
*N_s_*	Number of turns for secondary winding	*k_req_*	Required sensitivity
*d*	Gap between primary and secondary winding	*k_o_*	Sensitivity coefficient of sinh function circuit
*L_c_*	Length of LVDT moving core	*k*	Boltzmann’s constant
*L_a_*	Physical length of LVDT	*q*	Electron charge
*r_c_*	Radius of LVDT moving core	*T*	Absolute temperature, in degrees Kelvin
*v_ex_*	Excitation signal	*r_x_*	Intrinsic resistance at port X of CFOA
*V_P_*	Peak amplitude of excitation signal	*gm_c_*	Transconductance at port X of CFOA
*V_T_*	Thermal voltage	*gm*	Transconductance of OTA
